# Unveiling the presence and genotypic diversity of *Giardia duodenalis* on large-scale sheep farms: insights from the Henan and Ningxia Regions, China

**DOI:** 10.1186/s13071-024-06390-7

**Published:** 2024-07-19

**Authors:** Qianming Zhao, Xiaodong Ning, Zhiguang Yue, Fuchun Jian, Dongliang Li, Jiashu Lang, Shunli Lu, Changshen Ning

**Affiliations:** 1 College of Veterinary Medicine, Henan Agricultural University, Zhengzhou, 450046 Henan People’s Republic of China; 2 Henan Vocational College of Applied Technology, Zhengzhou, 450042 Henan People’s Republic of China; 3 Henan Anjin Biotechnology CO., LTD, Zhengzhou, 450011 Henan People’s Republic of China

**Keywords:** *Giardia duodenalis*, Multilocus genotype, MLG, Large-scale sheep farm, Public health

## Abstract

**Background:**

The parasitic protozoan *Giardia duodenalis* is an important cause of diarrheal disease in humans and animals that can be spread by fecal–oral transmission through water and the environment, posing a challenge to public health and animal husbandry. Little is known about its impact on large-scale sheep farms in China. In this study we investigated *G. duodenalis* infection of sheep and contamination of the environment in large-scale sheep farms in two regions of China, Henan and Ningxia.

**Methods:**

A total of 528 fecal samples, 402 environmental samples and 30 water samples were collected from seven large-scale sheep farms, and 88 fecal samples and 13 environmental samples were collected from 12 backyard farms. The presence of *G. duodenalis* was detected by targeting the β-giardin (*bg*) gene, and the assemblage and multilocus genotype of *G. duodenalis* were investigated by analyzing three genes: *bg*, glutamate dehydrogenase (*gdh*) and triphosphate isomerase (*tpi*).

**Results:**

The overall *G. duodenalis* detection rate was 7.8%, 1.4% and 23.3% in fecal, environmental and water samples, respectively. On the large-scale sheep farms tested, the infection rate of sheep in Henan (13.8%) was found to be significantly higher than that of sheep in Ningxia (4.2%) (*P* < 0.05). However, the difference between the rates of environmental pollution in Henan (1.9%) and Ningxia (1.0%) was not significant (*P* > 0.05). Investigations of sheep at different physiological stages revealed that late pregnancy ewes showed the lowest infection rate (1.7%) and that young lambs exhibited the highest (18.8%). Genetic analysis identified *G. duodenalis* belonging to two assemblages, A and E, with assemblage E being dominant. A total of 27 multilocus genotypes were identified for members of assemblage E.

**Conclusions:**

The results suggest that *G. duodenalis* is prevalent on large-scale sheep farms in Henan and Ningxia, China, and that there is a risk of environmental contamination. This study is the first comprehensive examination of the presence of *G. duodenalis* on large-scale sheep farms in China. Challenges posed by *G. duodenalis* to sheep farms need to be addressed proactively to ensure public health safety.

**Graphical Abstract:**

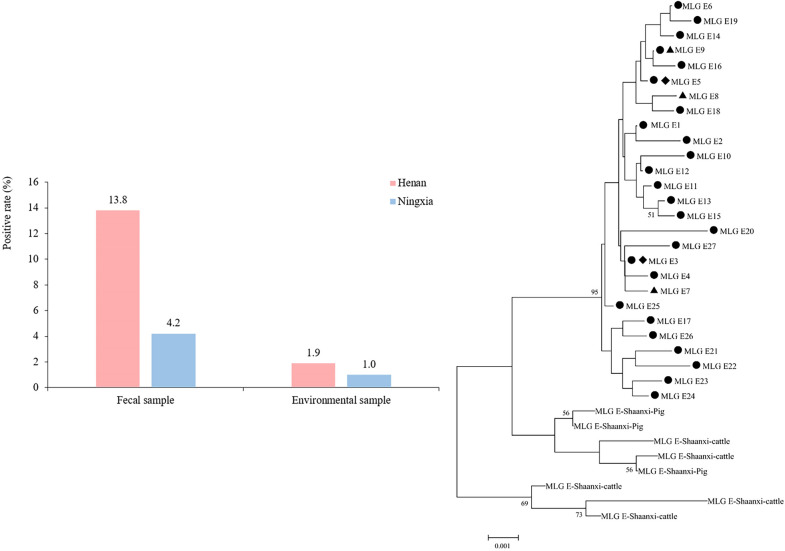

## Background

*Giardia duodenalis*, also known as *Giardia lamblia* and *Giardia intestinalis*, is a parasitic protozoan with global distribution. This pathogen is a primary contributor to diarrheal illness and can cause various symptoms, including nausea, vomiting and dehydration [1]. Severe *G. duodenalis* infections can result in death of the host [2]. While *G. duodenalis* infections may be asymptomatic, research indicates that they can lead to developmental delays in young animals and hinder reproductive capabilities in adult ones [3]. Furthermore, *G. duodenalis* has been linked with arthritis and irritable bowel syndrome in humans [4, 5].

*Giardia duodenalis* is categorized into eight assemblage types (assemblages A–H), with each assemblage having its own distinct host range. Assemblages A and B exhibit the widest host spectrum and are recognized as typical zoonotic assemblages capable of infecting humans and the majority of mammals [6]. The remaining assemblages are relatively host-specific; however, the host specificity is not always absolute. Although assemblages C-H are usually associated with hosts other than humans, assemblages C [7], E [8, 9] and F [10] have been documented in humans. Sheep play a significant role as hosts of *G. duodenalis*, with infection rates varying from 0 to 89.17% [11, 12]. In sheep, assemblage E is the predominant type in sheep, with assemblages A and B also commonly detected [13, 14]. Sheep are recognized as a potential source for the transmission of *G. duodenalis* infection to humans [14]. To better assess the zoonotic transmission of giardiasis and to differentiate mixed infections of assemblages, high-resolution multilocus genotyping analysis has been widely used to characterize *G. duodenalis* isolates from humans and animals by sequencing a number of genes with intrapopulation variants, including the genes for β-giardin (*bg*), glutamate dehydrogenase (*gdh*), and triosephosphate isomerase (*tpi*) [15, 16].

During the peak of infection, ruminants can excrete 10^6^ infective cysts per gram of feces, which are readily infectious upon excretion [17]. The cysts can remain infectious for several months in a suitable environment, leading to rapid accumulation in the high-density rearing environment of large-scale sheep farms. Healthy hosts primarily contract *G. duodenalis* through the fecal–oral route, which can occur from consuming contaminated water or food, or through direct contact with infected animals [6, 18]. *Giardia duodenalis* is well-suited for rapid environmental transmission in ruminants. While direct evidence of *G. duodenalis* transmission through contaminated environments is limited, evidence is accumulating that does suggest the spread of this parasite through a contaminated environment [19–21].

China is a significant sheep farming nation and has actively promoted large-scale sheep farming to advance agricultural modernization. However, high-density farming creates conditions conducive to *G. duodenalis* outbreaks. While many sheep infected with *G. duodenalis* do not display clinical symptoms, they still shed infectious *G. duodenalis* cysts into the environment. Given that exposure to these cysts through contaminated water and food is the primary mode of *G. duodenalis* transmission to animals and humans, the presence of these cysts on sheep farms could pose a threat to the surrounding environment and the health of individuals residing nearby.

The objective of this study was to investigate the prevalence and genetic diversity of *G. duodenalis* in large-scale sheep farms in Henan Province (Henan) and the Ningxia Hui Autonomous Region (Ningxia), China. Specifically, the study objectives were: (i) to determine the prevalence of infection in sheep at different physiological stages; (ii) to assess the contamination of the environment and water by *G. duodenalis* in large-scale sheep farms; and (iii) to analyze the genetic diversity of different isolates of *G. duodenalis* by using multilocus genotypes (MLGs) to gain insights into the epidemiology of the pathogen and the potential transmission of zoonotic diseases.

## Methods

### Sample collection

From September 2021 to March 2023 we conducted an observational study, collecting a total of 616 fresh fecal samples (sample size 5–30 g) by rectal sampling from sheep in two regions of China, Henan and Ningxia. Of the 616 samples collected, 528 were collected on seven large-scale sheep farms (sheep farms that adopt modern farming techniques and management methods for standardized production), of which 516 could be classified according to nine physiological stages: lactating lambs, weaning lambs, fattening lambs, young lambs, non-pregnant ewes, early-pregnancy ewes, late-pregnancy ewes, lactating ewes and breeding rams. A total of 26 fecal samples were collected from three of the seven large-scale sheep farms; the remaining four large-scale sheep farms were sampled in a proportional manner, with samples collected from 0.5% to 2% of sheep on each farm, resulting in a total of 502 samples. The remaining 88 samples were collected on 12 backyard farms (breeding in a private home backyard or small-scale setting, with each farm having < 300 sheep) (Table 1).

**Table 1:** Infection and environmental contamination of sheep with *Giardia duodenalis* in different regions and farm types in Henan and Ningxia regions, China

Sample source	Sample type	Sampling area	Percentage of positive samples (no. positive/no. sampled)	Genotype or subtype of *G. duodenalis* assemblage (*n*)^a^		
				*bg*	*gdh*	*tpi*
Large-scale sheep farms	Fecal sample	Henan	13.8 (37/269)	A (1), E (36)	E (24)	E (29)
		Ningxia	4.2 (11/259)	E (11)	E (10)	E (9)
	- Total		9.1 (48/528)	A (1), E (47)	E (34)	E (38)
	Environmental sample	Henan	1.9 (4/207)	E (4)	E (2)	E (3)
		Ningxia	1.0 (2/195)	E (2)	E (1)	E (2)
	- Total		1.5 (6/402)	E (6)	E (3)	E (5)
	Water sample	Henan	23.3 (7/30)	E (7)	E (3)	E (6)
Backyard breeding/small-scale farms	Fecal sample	Ningxia	0 (0/88)	–	–	–
	Environmental sample	Ningxia	0 (0/13)	–	–	–
Total	Fecal sample		7.8 (48/616)	A (1), E (47)	E (34)	E (38)
	Environmental sample		1.4 (6/415)	E (6)	E (3)	E (5)
	Water sample		23.3 (7/30)	E (7)	E (3)	E (6)

*bg* β-Giardin gene, *gdh* glutamate dehydrogenase gene, *tpi* triosephosphate isomerase gene

^a^*Giardia duodenalis* is categorized into eight assemblage types (assemblages A–H). The number in parentheses is the number of contaminated samples in which that assemblage was identified

Environmental samples (sample size 5–30 g) were collected randomly at the entrance of the building(s) used for sheep housing, inside the building(s), at the exit of the building(s) and in the aisles of the building(s). The collected samples were placed in clean plastic bags and numbered, and registration information recorded. A total of 415 environmental samples were collected, of which 402 were from large-scale sheep farms and 13 from places where backyard sheep rearing takes place (Table 1).

On a large sheep farm in Ruzhou, Henan Province, drinking water samples from two to four sheep were collected from pens at each physiological stage (30 samples in total). Each water sample (50 ml) was placed in a clean centrifuge tube, labeled with a number and registered (Table 1). All of the samples were labeled and stored, then transferred to the laboratory while preserving the cold chain; in the laboratory, the samples were kept at 4 °C until examination within 48 h. Should the allotted time be exceeded, a 2.5% solution of potassium dichromate was added to each sample and the samples stored at 4 °C until examination.

### DNA extraction and PCR amplification

DNA was extracted from each stool sample (approx. 200 mg) using a stool DNA extraction kit (Omega Bio-Tek Inc., Norcross, GA, USA) according to the manufacturer’s recommendations. The extracted DNA was stored at − 20 °C until PCR amplification. PCR amplification was first performed on all samples against the *bg* gene [22] to detect the presence of *G. duodenalis* and to determine the assemblage type. *Bg*-positive samples were then subjected to PCR amplification based on the *gdh* and *tpi* genes [23, 24] to determine the MLG. Two sets of primers specific for assemblage A and assemblage E at the *tpi* gene were used for amplification to enhance the identification of mixed infections involving different assemblages of *G. duodenalis* [25].

### Sequencing and sequence analysis

All PCR amplification products with the appropriate fragment size were purified and sequenced by SinoGenoMax Co. Ltd. (Beijing, China). Bidirectional sequencing was used to ensure sequence accuracy. The sequences were proofread for DNA peak patterns using SeqMan in DNASTAR (DNASTAR, Inc., Madison, WI, USA; http://www.dnastar.com/). The Clustal X v2.0 software (http://www.clustal.org/) and data from GenBank were used to compare and identify the manually spliced sequences. The amplification results for the *bg*, *gdh* and *tpi* genes were analyzed to reveal the genetic diversity of *G. duodenalis*.

To examine the relationships between various isolates and uncover the genetic diversity of *G. duodenalis*, we used MEGA 7.0 software (https://www.megasoftware.net/) to construct a phylogenetic tree based on the neighbor-joining method [26]. Gene sequences were concatenated (*bg–tpi–gdh*), and the Tamura-Nei model was chosen for analysis [27]. The reliability of the evolutionary tree was analyzed by performing 1000 replications using the bootstrap method for phylogenetic analysis.

### Statistical analysis

Infection rates were analyzed by region and physiological stage using Chi-square* (χ*^2^) analysis and SPSS software v26 (SPSS–IBM Corp., Armonk, NY, USA). *P* < 0.05 was considered statistically significant.

## Results

### *Giardia duodenalis* infection in sheep and environmental contamination

*Giardia duodenalis* was detected in 48 (7.8%; 95% confidence interval [CI] 5.7–9.9%) fecal samples, six (1.5%; 95% CI 0.3–2.7%) environmental samples and seven (23.3%; 95% CI 7.3–39.4%) water samples (Table 1). A total of 528 fecal samples from the large-scale sheep farms were analyzed, with an overall infection rate of 9.1% (48/528; 95% CI 6.6–11.6%) (Table 1). The infection rate on sheep farms in Henan was notably higher (13.8% [37/26]; 95% CI 9.6–17.9%) than that in Ningxia (4.2% [11/259]; 95% CI 1.8–6.7%; *χ*^2^ = 14.432, *df* = 1, *P* < 0.05 (Fig. 1). A total of 402 environmental samples from large-scale sheep farms were examined, with an overall detection rate of 1.5% (6/402; 95% CI 0.3–2.7%). The detection rate on the farms in Henan was 1.9% (4/207; 95% CI 0.0–3.8%), which was slightly higher than that on the farms in Ningxia (1.0% [2/195]; 95% CI 0.0–2.5%; *χ*^2^ = 0.561, *df* = 1, *P* > 0.05). Of the 30 water samples analyzed, seven (23.3%; 95% CI 7.3–39.4%), which were all collected from large-scale farms in the Ruzhou area of Henan, tested positive for *G. duodenalis*. *Giardia duodenalis* was not detected in any of the 88 fecal samples or 13 environmental samples tested from sites of backyard sheep rearing (Table 1).

**﻿Fig. 1:**
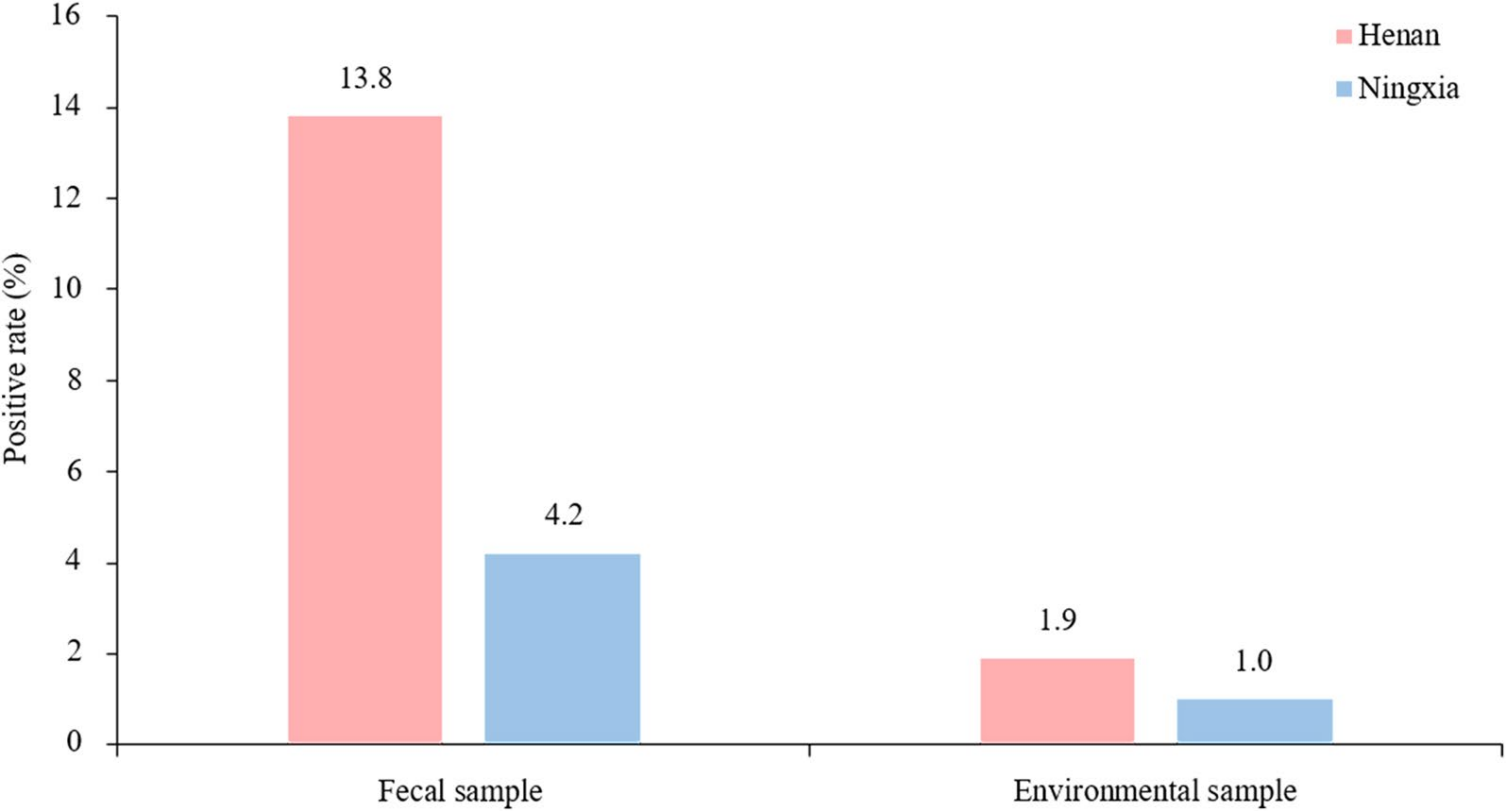
Infection and environmental contamination of sheep with *Giardia duodenalis* on large-scale sheep farms in the Henan and Ningxia regions, China. Numbers above bars are the infection rates

Four breeds of sheep were examined in this study on large-scale sheep farms, with the highest infection rate of 15.2% (39/257; 95% CI 10.8–19.6%) registered in Hu sheep. This infection rate was significantly higher than the infection rate of 3.0% (7/236; 95% CI 0.8–5.1%) registered in Tan sheep.

**Fig. 2:**
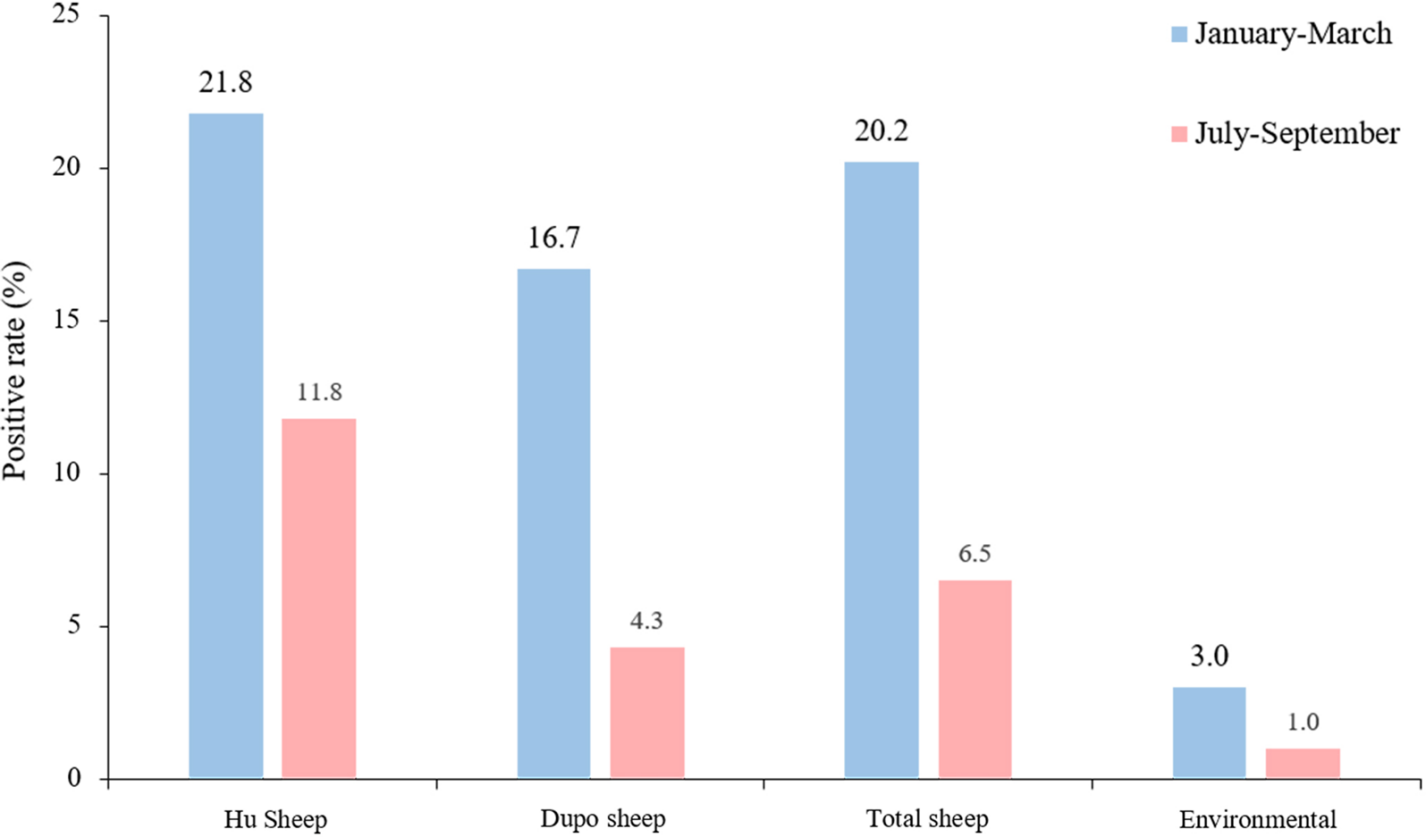
*Giardia duodenalis* infection rates and environmental contamination of sheep on large-scale sheep farms during different time periods. Numbers above bars are the infection rates

Samples from large-scale sheep farms were collected over two time periods: January to March (winter to early spring, when temperatures are relatively lower) and July to September (from summer to early autumn, when temperatures are relatively higher). Fecal testing revealed that the prevalence of *G. duodenalis* infection in sheep was 20.2% (20/99; 95% CI 12.2–18.3%) during the January–March period, which was significantly higher than the 6.5% (28/429; 95% CI 4.2–8.9%) registered during the July–September period (*χ*^2^ = 19.797, *df* = 1, *P* < 0.05). For environmental samples, the detection rate was 3.0% (3/99; 95% CI 0.0–6.5%) in the January-March period, which was slightly higher than that of 1.0% (3/303; 95% CI 0.0–2.1%) registered in the July–September period, but the difference was not statistically significant (*χ*^2^ = 2.113, *df* = 1, *P* > 0.05) (Table 2; Fig. 2).

**Table 2:** *Giardia duodenalis* infection in different breeds of sheep and positivity of environmental samples at different sampling times

Sampling time	Sample source	Percentage of positive samples (no. positive/no. sampled)	Genotype or subtype of *G. duodenalis* assemblage (*bg* gene)^a^
January-March	Hu sheep	21.8 (19/87)	E (19)
	Yuxi fat-tailed sheep	0.0 (0/6)	–
	Dupo sheep	16.7 (1/6)	A (1)
	Total sheep	20.2 (20/99)	A (1) E(19)
	Environmental	3.0 (3/99)	E (3)
July–September	Tan sheep	3.0 (7/236)	E (7)
	Hu sheep	11.8 (20/170)	E (20)
	Dupo sheep	4.3 (½3)	E (1)
	Total sheep	6.5 (28/429)	E (28)
	Environmental	1.0 (3/303)	E (3)
	Water	23.3 (7/30)	E ( (7)
Total	Hu sheep	15.2 (39/257)	E (39)
	Tan sheep	3.0 (7/236)	E (7)
	Dupo sheep	6.9 (2/29)	A (1) E (1)
	Yuxi fat-tailed sheep	0.0 (0/6)	–
	Environmental	1.5 (6/402)	E (6)
	Water	23.3 (7/30)	E (7)

*bg* β-Glutamate dehydrogenase gene

^a^The number in parentheses is the number of contaminated samples in which that assemblage was identified in the *bg* gene

After analyzing 516 fecal samples from sheep at distinct physiological stages on large-scale sheep farms, variations in infection rates were observed among the physiological stages. Notably, young lambs exhibited the highest infection rate, 18.8% (13/69; 95% CI 9.4–28.3%), followed by weaning lambs (17.3% [9/52]; 95% CI 6.7–27.9%). Late-pregnancy ewes displayed the lowest infection rate (1.7% [1/58]; 95% CI 0.0–5.2%). Infection rates in sheep at other physiological stages ranged from 5.3% to 10.0% (Table 3).

**Table 3:** *Giardia duodenalis* infection in sheep at different physiological stages on large-scale sheep farms in the Henan and Ningxia regions, China

Physiological stage	Percentage of positive samples (no. positive/no. sampled)	*P*-value	Genotype or subtype of *G. duodenalis* assemblage (*n*)^a^		
			*bg*	*gdh*	*tpi*
Lactating lambs	10.0 (3/30)	0.077	E (3)	E (3)	E (3)
Weaning lambs	17.3 (9/52)	0.005	E (9)	E (3)	E (5)
Fattening lambs	6.1 (5/82)	0.208	E (5)	E (5)	E (5)
Young lambs	18.8 (13/69)	0.002	E (13)	E (9)	E (11)
Non-pregnant ewes	7.5 (4/53)	0.140	E (4)	E (3)	E (3)
Early-pregnancy ewes	7.3 (4/55)	0.152	E (4)	E (2)	E (2)
Late-pregnancy ewes	1.7 (1/58)	Reference group	E (1)	E (1)	E (1)
Lactating ewes	5.3 (3/57)	0.300	E (3)	E (2)	E (2)
Breeding rams	8.3 (5/60)	0.102	A (1), E (4)	E (5)	E (5)
Total	9.1 (47/516)		A (1), E (46)	E (33)	E (37)

*bg* β-Giardin gene, *gdh* glutamate dehydrogenase gene, *tpi* triosephosphate isomerase gene

^a^*Giardia duodenalis* is categorized into eight assemblage types (assemblages A–H). The number in parentheses is the number of contaminated samples in which that assemblage was identified

### *Giardia duodenalis* assemblage distribution

A total of 61 samples positive for the *bg* gene were identified in nested PCR analysis targeting *G. duodenalis* (48 fecal samples, 6 environmental samples and 7 water samples). Among these, a human–animal co-infection with assemblage A was identified in one fecal sample, while the remaining samples belonged to assemblage E.

In the 61 samples identified as positive through analysis of the *bg* gene, the *gdh* gene was also analyzed. The results of this *gdh* gene analysis showed that 34 fecal samples, three environmental samples and three water samples were classified as belonging to assemblage E. A similar analysis of these 61 samples identified as positive based on analysis of the *bg* gene was conducted using the *tpi* gene. In the 48 fecal samples, assemblage E was recorded in 38 samples, along with five occurrences in environmental samples and six in water samples. Importantly, analysis of neither the *gdh* nor the *tpi* genes indicated the presence of assemblage A.

### Subtypes of assemblages A and E

From among all 1060 samples, a total of 61, 49 and 40 sequences were obtained for the *bg*, *tpi* and *gdh* genes, respectively (Table 1). Using the sequence with GenBank accession number KT922248 as the reference sequence for the *bg* gene, the 60 sequences from assemblage E were identified to belong to 12 subtypes, of which three were newly discovered (PP934567–PP934569) (Table 4). Employing the reference sequence MF095054, six subtypes were identified from 49 *tpi* gene sequences, with one new subtype discovered (PP507056) (Table 5). Using KY711410 as the reference sequence, the 40 *gdh* gene sequences were divided into 12 subtypes, revealing seven new subtypes (PP934570–PP934574, PP507057–PP507058) (Table 6).

**Table 4:** Intra-assemblage substitutions in *Giardia duodenalis* assemblage E in the β-giardin gene loci

Subtype	Nucleotide at position										GenBank ID	Number of positive samples		
												Fecal sample	Environmental sample	Water sample
*bg*	49	53	65	107	170	383	413	463	472	488				
Reference sequence	T	C	C	C	A	C	T	C	C	C	KT922248			
E1	–	–	–	–	–	–	–	–	–	–	KY633466	13	2	4
E2	–	–	–	–	–	–	C	–	–	–	MK610388	13	4	3
E3	–	-	T	–	–	–	C	–	–	–	KP635114	5	0	0
E4	–	T	–	–	–	–	–	–	–	–	OP142426	4	0	0
E5	–	–	T	–	–	T	C	–	–	–	KP635098	3	0	0
E6	–	–	T	T	G	–	–	–	–	–	MK610379	3	0	0
E7	–	–	T	–	G	–	C	–	–	T	KY432834	1	0	0
E8	–	–	T	–	G	–	C	–	–	–	KY633471	1	0	0
E9	–	–	T	–	–	–	–	–	–	–	KP635113	1	0	0
E10	C	–	T	–	–	–	C	–	–	–	PP934567	1	0	0
E11	–	–	–	–	–	–	–	–	–	T	PP934568	1	0	0
E12	–	–	T	–	–	T	C	T	A	–	PP934569	1	0	0

*bg* β-Giardin gene

**Table 5:** Intra-assemblage substitutions in assemblage E in the triosephosphate isomerase gene loci

Subtype	Nucleotide at position					GenBank ID	Number of positive samples		
							Fecal sample	Environmental sample	Water sample
*tpi*	91	158	168	262	400				
Reference sequence	G	A	C	T	G	MF095054			
E1	–	–	–	–	–	MF095054	1	0	2
E2	A	–	–	–	–	MH230888	18	1	2
E3	A	–	–	–	A	MK473862	6	0	0
E4	A	–	–	C	–	MK442915	9	2	1
E5	A	–	T	C	–	OP156823	4	1	1
E6		G	–	–	–	PP507056	0	1	0

*tpi* Triosephosphate isomerase gene

**Table 6:** Intra-assemblage substitutions in assemblage E in the glutamate dehydrogenase gene loci

Subtype	Nucleotide at position											GenBank ID	Number of positive samples		
													Fecal sample	Environmental sample	Water sample
*gdh*	21	129	168	243	264	297	318	333	366	384	396				
Reference sequence	C	C	G	C	C	C	C	A	G	C	C	KY711410			
E1	–	–	–	–	–	–	–	–	–	–	–	KY711410	10	0	0
E2	–	–	A	–	–	–	–	–	–	–	–	AB692774	15	2	2
E3	–	–	–	–	–	–	A	–	–	–	–	KP635107	1	0	0
E4	–	–	A	T	–	–	–	–	–	–	–	MT123526	1	0	0
E5	–	–	A	–	–	–	–	G	–	T	–	OP142431	1	0	0
E6	–	–	A	–	–	–	–	–	A	–	–	PP934570	1	0	0
E7	–	–	A	–	–	–	A	–	–	–	–	PP934571	1	0	0
E8	–	–		T	–	T	–	–	–	–	–	PP934572	1	0	0
E9	–	–		–	–	–	–	–	–	T	–	PP934573	1	0	0
E10	–	–		–	–	–	–	–	–	–	T	PP934574	0	1	0
E11	T	–	A	–	–	–	–	–	–	–	–	PP507057	2	0	0
E12	–	–	A	–	–	–	–	–	–	T	–	PP507058	0	0	1

*gdh* Glutamate dehydrogenase gene

For the *bg* gene, one sequence belonging to assemblage A was identified that exhibited 100% homology with reference sequence MN629930.

### Nucleotide sequence accession numbers

Representative nucleotide sequences from this study have been deposited in the NCBI GenBank database, with accession numbers PP934567–PP934569 for the *bg* gene, PP934570–PP934574 and PP507057–PP507058 for the *gdh* gene and PP507056 for the *tpi* gene.

### Multilocus genotyping

**Fig. 3:**
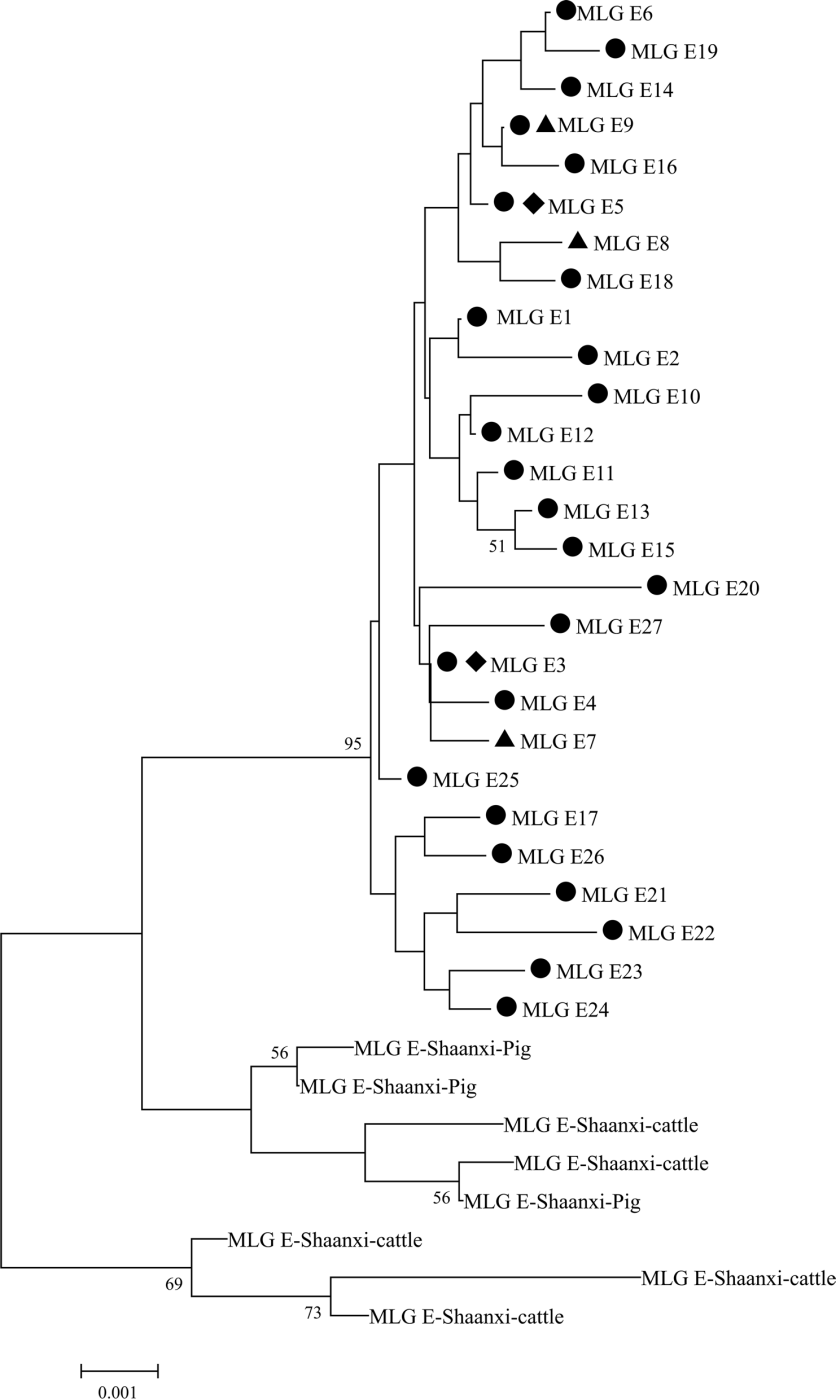
Phylogenetic relationships among *Giardia duodenalis* MLGs. The phylogenetic tree was constructed using a concatenated dataset of *bg*, *tpi* and *gdh* gene sequences and neighbor-joining analysis with the Tamura-Nei model. Bootstrap values > 50% from 1000 replicates are shown at nodes. MLGs marked with black circles indicate sequences obtained from fecal samples in this study; black triangles indicate sequences obtained from environmental samples; and black diamonds indicate sequences obtained from water samples. *bg*, β-Giardin gene; *gdh*, glutamate dehydrogenase gene; MLG, multilocus genotype; *tpi*, triosephosphate isomerase gene

A total of 38 samples, including 33 fecal samples, three environmental samples and two water samples exhibited amplification of all three genes (i.e., *bg*, *gdh*, and *tpi*) simultaneously. Among the 33 fecal samples, 32 had sequences belonging to assemblage E at all three gene loci, yielding 25 distinct assemblage E MLGs (MLG1–MLG6, MLG9–MLG27), while one sample showed mixed characteristics of assemblage A and assemblage E. The three environmental samples formed three assemblage E MLGs (MLG7–9), and the two water samples formed two assemblage E MLGs (MLG3 and MLG5) (Table 7; Fig. 3). All of the assemblages of E MLGs obtained in this study have close affinities.

**Table 7:** Multilocus sequence genotyping of *Giardia duodenalis* in sheep in this study using the β-giardin, glutamate dehydrogenase and triosephosphate isomerase genes

Isolate/specimen ID^a^	Genotype			MLG
	*bg*	*tpi*	*gdh*	
415	E1	E2	E1	MLG E1
1019	E1	E2	E8	MLG E2
1155, 1980b	E1	E2	E2	MLG E3
1358	E1	E2	E6	MLG E4
1821, 1830, 1831, 1834, 1981b	E1	E4	E2	MLG E5
1832	E1	E5	E2	MLG E6
1934a	E1	E6	E2	MLG E7
1383a	E1	E4	E10	MLG E8
1017, 1904a	E2	E4	E2	MLG E9
1079	E2	E1	E4	MLG E10
1335	E2	E2	E1	MLG E11
1369, 1826, 1828	E2	E2	E2	MLG E12
1372	E2	E2	E7	MLG E13
1805	E2	E5	E2	MLG E14
1806	E2	E2	E3	MLG E15
1823	E2	E4	E11	MLG E16
1864	E3	E2	E11	MLG E17
1147	E4	E4	E1	MLG E18
1807	E4	E5	E2	MLG E19
1822	E4	E2	E5	MLG E20
417, 1052, 1068	E5	E3	E1	MLG E21
1547	E6	E3	E1	MLG E22
1025	E7	E2	E2	MLG E23
1560	E8	E2	E1	MLG E24
1521	E9	E2	E2	MLG E25
1151	E10	E2	E2	MLG E26
1096	E11	E3	E1	MLG E27

*bg* β-Giardin gene, *gdh* glutamate dehydrogenase gene,* MLG* multilocus genotype, *tpi* triosephosphate isomerase gene

^a^Isolate/specimen ID followed by a lowercase 'a' is an environmental sample; isolate/specimen ID followed by 'b' is a water sample

## Discussion

The prevalence of *G. duodenalis* infection in sheep in this study was 7.8% (48/616) by testing the *bg* gene. This infection rate is higher than that of sheep in Inner Mongolia (3.4%, 27/797) [26] and lower than that of sheep in Jiangsu (30.0%, 36/120) [28], but comparable to that in Xinjiang (7.5%, 24/318) [29]. The infection rate of Hu sheep in this study was found to be lower (15.2%) than that reported for Hu sheep on a sheep farm in Henan Province (17.9%, 81/474) [30]. Also, the infection rate of beach sheep in this study was observed to be 3.0%, which is lower than that reported in previous surveys in Ningxia (14.5%, 147/1014) [31]. These discrepancies indicate that sample size, sampling time and—potentially—environmental conditions may be pivotal in determining the infection rate of an animal population. Future comparisons and analyses will assist in elucidating the specific effects of these variables.

The results of this study demonstrated that the prevalence of *G. duodenalis* infection in sheep was significantly higher during the months of January to March (20.2%) than during the months of July to September (6.5%; *P* < 0.05). The analysis of environmental samples similarly revealed that the detection rate of *G. duodenalis* was higher during the months of January to March (3.0%) than during the months of July to September (1.0%; *P* > 0.05). These results may indicate that environmental factors play a role in the harborage of *G. duodenalis* in certain seasons [30] and suggest that seasonal variation may be an important factor regulating the dynamics of *G. duodenalis* infections.

Based on information in the Baidu Encyclopedia (https://baike.baidu.com/), the Henan region has a temperate monsoon climate with an annual temperature that ranges approximately from 10.5 °C to 16.7 °C and annual precipitation that ranges approximately from 407.7 to 1295.8 mm. The Ningxia region has a temperate continental climate with an annual temperature that ranges approximately from 6.3 °C to 11.4 °C and annual precipitation that ranges from 164.1 to 739.4 mm. Studies have suggested that *G. duodenalis* cysts may exhibit higher activity in warm and humid environments, thereby increasing the chance that they infect a host and promoting disease development [13, 33]. These factors could potentially contribute to the higher infection rate observed in sheep from large-scale farms in Henan (13.8%) compared with those in Ningxia (4.2%). Additionally, the positivity rate of *G. duodenalis* in environmental samples from Henan (1.9%) was higher than that in environmental samples from Ningxia (1.0%), which seems to further support the aforementioned hypothesis.

While backyard farms may pose a greater risk in terms of the transmission of wildlife-borne diseases to domestic animals, intensive farming conditions can lead to the emergence and expansion of epidemics [34]. The study conducted in Ningxia revealed a significantly higher infection rate of *G. duodenalis* in sheep on large-scale farms (4.2%) compared with backyard farms (0.0%; *P* < 0.05). Additionally, the prevalence of *G. duodenalis* positivity in environmental samples from large-scale sheep farms (1.0%) exceeded that in environmental samples from backyard farms (0.0%). The susceptibility of large-scale farming to epidemics may stem from the scale and density of breeding, posing greater health challenges. Large-scale sheep farms typically employ centralized manure disposal methods, which can impact the survival and transmission of intestinal parasites such as *G. duodenalis.* It is crucial to take this into account when assessing the potential risks associated with farms of this type.

Multiple studies have previously shown a negative correlation between the prevalence of *G. duodenalis* infection and age [32, 35, 36], a result that aligns with the findings of the present study. In the current study, the prevalence of *G. duodenalis* infection was higher in immature sheep than in adult sheep across all physiological stages except for fattening lambs. The self-limiting nature of *G. duodenalis* infection and the intermittent excretion of cysts may account for the lower prevalence of *G. duodenalis* infection in fattening lambs compared with young and weaning lambs in the current study [37, 38]. The lowest infection rate in this study was observed in late-pregnancy ewes, likely because of immune system adaptations that occur during this period to accommodate the fetus and prevent rejection [39]. These immune system changes may enhance the resistance of ewes to certain diseases in late pregnancy.

*Giardia duodenalis* can lead to giardiosis, which is transmitted through contact and consumption of contaminated water and soil [40]. Although the positivity rate in environmental samples in this study was only 1.5%, this rate indicates that *G. duodenalis* cysts are present in the buildings housing sheep and that the transmission cycle of *Giardia* is likely perpetuated through the feeding behavior of other sheep. Our results indicate that contamination of water by *G. duodenalis* is more severe than contamination of the environment, as demonstrated by a positivity rate of 23.3% in 30 samples of sheep drinking water collected from a large-scale farm in Henan Province. This finding suggests that sheep may be more susceptible to *G. duodenalis* infection through the drinking water route, emphasizing the need for stricter control and preventive measures to reduce the risk of infection. The presence of *G. duodenalis* in the feeding environment poses a health threat to sheep and could potentially lead to transmission to humans through contaminated food or water [41]. The results of this analysis indicate that biosecurity strategies should be actively pursued in large-scale sheep farms, including providing a supply of clean drinking water, optimizing sheep manure management and providing staff with the necessary health and safety training. These measures aim to reduce the spread of disease, thereby improving the health of the farming industry and indirectly protecting overall human health and reducing potential risks.

Genetic variants of *G. duodenalis* have been documented in sheep globally, with a total of five assemblages (A, B, C, D and E) identified so far [42–44]. However, in China, only three assemblages (A, B and E) have been detected in sheep [12]. The present study validates these previous findings in China, revealing only one case of assemblage A (detected via analysis of the *bg* gene), while the remaining cases were identified as belonging to assemblage E. Assemblage E is commonly linked to hoofed animals [45]; however, cases of human infection with assemblage E have been reported in Brazil [8], Australia [9], Egypt [46, 47] and New Zealand [48]. Sporadic reports suggest that this assemblage can infect humans, highlighting a potential zoonotic public health risk. The high prevalence of zoonotic *G. duodenalis* assemblages on large-scale sheep farms may indicate that sheep farms are important reservoirs of human* Giardia*. These findings suggest that strict hygiene practices and regular surveillance for *G. duodenalis* on large-scale sheep farms may be required to prevent potential outbreaks.

To obtain a more comprehensive understanding of mixed infections in sheep by different assemblages of *G. duodenalis*, we chose two sets of primers, specific for assemblage A and assemblage E, respectively, for amplification of the *tpi* gene in the current study [25]. However, no sequences of the *tpi* gene associated with assemblage A were acquired, indicating that mixed infections of sheep with both assemblage A and assemblage E were less common in this study than in previous studies [30]. To delve deeper into the genetic variation of assemblage E in *G. duodenalis*, we employed a multilocus genotyping tool to simultaneously amplify three genes in 37 samples that were positive for assemblage E based on analysis of the *bg* gene. A tandem sequence (*bg–tpi–gdh*)-based evolutionary tree was constructed, resulting in the identification of 27 new MLGs (Table 7). The findings indicate that assemblage E exhibits high subtype diversity and genetic variation.

Due to the limitations of the sampling and geographical scope (restricted to large-scale sheep farms in Henan and Ningxia), the representativeness of this study is constrained and the findings do not fully reflect the national situation. The *bg* locus was employed to detect *G. duodenalis*, and although this locus is a common occurrence, PCR efficiency may influence the accuracy of the results. To enhance understanding of the impact of this pathogen on large-scale sheep farms and public health, future studies should utilize random sampling methods and ensure broader geographic coverage. The inclusion of different seasons and different farm management practices would provide a more comprehensive understanding of *G. duodenalis* dynamics. In addition, incorporating advanced molecular techniques could improve detection sensitivity and provide greater insight into the epidemiology of *G. duodenalis*.

## Conclusions

In this study, we conducted an epidemiological investigation of *G. duodenalis* in sheep, the environment and drinking water on selected large-scale sheep farms in Henan Province and the Ningxia Hui Autonomous Region, China. The findings reveal the widespread presence of *G. duodenalis* on these large-scale sheep farms. Phylogenetic analyses demonstrated a close relationship among all of the identified isolates of *G. duodenalis*, underscoring the importance of enhanced detection and surveillance on large-scale sheep farms. It is imperative to prioritize the maintenance of clean and hygienic sheep farming environments and drinking water to prevent environmental parasite contamination and pathogen transmission.

Future research efforts could focus on evaluating the effectiveness of specific control measures, exploring alternative approaches to parasite management and investigating the potential public health impact of *G. duodenalis* beyond the farm environment. By addressing these areas, we can develop more comprehensive strategies to effectively control *G. duodenalis* and similar pathogens.

## Data Availability

All data supporting the conclusions of this study are available in the manuscript. Representative nucleotide sequences from this study have been deposited in the NCBI GenBank database, with accession numbers PP934567–PP934569 for the *bg* gene, PP934570–PP934574 and PP507057–PP507058 for the *gdh* gene and PP507056 for the *tpi* gene.
